# A clinical risk score enables early prediction of dissatisfaction 1 year after total knee arthroplasty

**DOI:** 10.1002/ksa.12277

**Published:** 2024-05-26

**Authors:** André Strahl, Maximilian M. Delsmann, Alexander Simon, Christian Ries, Tim Rolvien, Frank Timo Beil

**Affiliations:** ^1^ Department of Trauma and Orthopaedic Surgery, Division of Orthopaedics University Medical Center Hamburg‐Eppendorf Hamburg Germany

**Keywords:** multiple regression, prediction model, risk score, satisfaction, total knee arthroplasty, total knee replacement

## Abstract

**Purpose:**

Dissatisfaction after total knee arthroplasty (TKA) is a prevalent and clinically relevant problem that affects approximately 10%–20% of patients. The aim of this study is to identify factors associated with dissatisfaction 1 year after TKA.

**Methods:**

A total of 236 patients undergoing TKA were included in this prospective cohort study. Demographic data, preoperative clinical parameters (e.g., axial alignment, osteoarthritis severity) and patient‐reported outcome measures (PROMs) were collected preoperatively, at 1 month and 1 year after TKA, encompassing the Knee Society Score (KSS) and Knee injury and Osteoarthritis Outcome Score (KOOS). The primary outcome was dissatisfaction 1 year after TKA, defined as ≤20 points on the KSS satisfaction scale. A risk score based on multiple regression and area under the curve (AUC) analyses was calculated to predict dissatisfaction.

**Results:**

One year after TKA, 16% of the patients were dissatisfied. Dissatisfied patients were significantly younger (*p* = 0.023) and had a higher body mass index (BMI) (*p* = 0.007). No differences were observed in preoperative objective (*p* = 0.903) and functional KSS (*p* = 0.346), pain (*p* = 0.306), osteoarthritis severity (*p* = 0.358), axial knee alignment (*p* = 0.984) or psychological distress (*p* = 0.138). The likelihood of dissatisfaction at 1 year was 3.0, 4.0, 7.4, 4.3 and 2.8 times higher amongst patients aged <63.5 years, with a BMI > 30.1 kg/m^2^, a KOOS_Pain_ < 50%, a KSS_Function_ < 42 points and a KSS_Expectation_ < 9 points (all at 1 month), respectively. Using these variables, a risk score with a maximum of 7 points was developed, demonstrating a high predictive value for dissatisfaction (AUC: 0.792 [95% confidence interval: 0.700–0.884], *p* < 0.001).

**Conclusion:**

Dissatisfaction 1 year after TKA can be predicted by a weighted risk score that includes patient age, BMI, pain, subjective functionality and unmet expectation 1 month postoperatively. Using the risk score, early detection of dissatisfaction has the potential to enable targeted interventions and improve patients' quality of life.

**Level of Evidence:**

Level II, Prognostic study.

**Abbreviations**
ADL: activities of daily living
ANOVA: analysis of variance
ASA: American Society of Anesthesiologists
AUC: area under the curve
BMI: body mass index
CI: confidence interval
HKA: hip–knee–ankle
KL: Kellgren–Lawrence
KOOS: Knee injury and Osteoarthritis Outcome Score
KSS: Knee Society Score
OR: odds ratio
PHQ: Patient Health Questionnaire
PROM: patient‐reported outcome measure
QoL: quality of life
ROC: receiver operating curves
ROM: range of motion
SD: standard deviation
SE: standard error
Sport/Rec: sport and recreation
TKA: total knee arthroplasty
TRIPOD: Transparent Reporting of a Multivariable Prediction Model for Individual Prognosis or Diagnosis
VAS: visual analogue scale

## INTRODUCTION

Patient satisfaction has become an increasingly important outcome measure after total knee arthroplasty (TKA) alongside standardised patient‐reported outcome measures (PROMs) [21, 26, 38]. The rate of dissatisfaction after TKA has been reported to range from 8% to 25% [4, 6, 23]. According to the estimates of a systematic review, the rate of dissatisfaction averages around 10% [13]. The prevalence of dissatisfaction varies due to different measurement methods used in previous studies, with only few validated satisfaction scores available [23]. Previous studies have employed different self‐administered satisfaction scales, including four‐item [7, 9, 37, 43] or five‐item [2, 15, 20, 25] Likert scales, numeric rating scales [19, 42, 45] or visual analogue scales (VASs) [29, 34, 46]. Additionally, some studies aggregated several single items to create a metric score [4, 8, 10]. In contrast, the use of a validated satisfaction scale, for example, obtained from the Knee Society Score (KSS) [40], has only been used in few studies [5, 34, 39]. Hence, the investigated predictors for dissatisfaction exhibit a high degree of diversity and variability.

The risk factors for dissatisfaction identified in a systematic review include patient age <65 years, low income, low osteoarthritis grade, preoperative depression or anxiety, complications, unmet expectations and persistent postoperative pain and stiffness [13]. Further studies have reported that patient satisfaction is negatively influenced by female sex [10], high body mass index (BMI) [9], high preoperative pain intensity [9], high absolute preoperative PROM scores [17], anterior tibial component slope and greater valgus angle of the femoral component [19], a higher number of nonorthopaedic comorbidities [7], only subclinical improvements in general life satisfaction [8], an American Society of Anesthesiologists grade >2 [25], low health literacy [39] and previous knee surgery [36, 42]. In general, patient satisfaction is influenced by several factors, some of which are beyond the control of the orthopaedic surgeon [15, 19].

Most studies that have examined the prediction of dissatisfaction have reported independent risk factors, but generally have not provided a clinical application in the form of a risk score. Consequently, these findings typically lack direct implications for clinical practice. Only Van Onsem et al. [41] have previously developed a score to predict satisfaction after TKA. However, this previous study was limited to a sample size of 113 patients and a follow‐up period of 3 months, which limits the informative value with regard to longer follow‐up periods. Addressing this knowledge gap, the purpose of this study was to develop a practical risk score to predict dissatisfaction 1 year after TKA. We hypothesised that certain demographic and clinical variables, both preoperatively and in the early postoperative period, are associated with an increased risk of dissatisfaction 1 year after TKA.

## METHODS

### Data collection and study population

**Figure 1:**
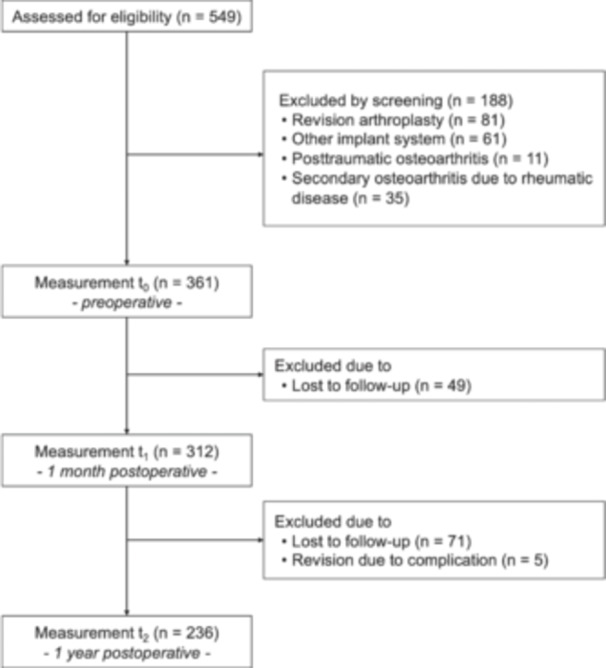
Flowchart of patients included in the study. Flowchart illustrating the inclusion and exclusion criteria for patient selection during the various measurement time points: preoperative assessment, 1 month and 1 year after total knee arthroplasty.

This was a single‐centre prospective cohort study including patients with end‐stage primary knee osteoarthritis undergoing TKA. The indication for surgery was made by an attending orthopaedic surgeon based on symptoms, clinical examination and radiographic findings. Clinical data were extracted from the medical records and supplemented by PROMs collected prospectively. Standardised surveys were obtained before admission, 1 month and 1 year after surgery. A total of 549 possible patients were initially screened for eligibility (Figure 1). Of those, we considered all patients undergoing primary TKA with an ultracongruent insert design and without patellar resurfacing (balanSys® Bicondylar, Mathys AG). Based on that, 188 patients were excluded because they initially presented for revision arthroplasty (*n* = 81), a different implant system was used (*n* = 61), posttraumatic osteoarthritis (*n* = 11) or secondary osteoarthritis due to a rheumatic disease (*n* = 35) was present. In addition, 49 patients were lost to follow‐up at 1 month and an additional 71 patients at 1 year postoperatively. Another five patients who had major complications requiring revision surgery during the follow‐up period were excluded, leaving 236 patients with complete data, including the 1‐year follow‐up, for final analysis. The medial parapatellar approach was used by a team of five attending surgeons to ensure a standardised technique and collaborative patient care. Cemented femoral and tibial implant fixation was uniformly used in all procedures.

### Clinical and PROMs

The KSS, one of the most widely used and extensively validated measures of TKA outcomes [1, 30, 33, 35], was assessed preoperatively, 1 month and 1 year postoperatively. It contains various items and subscales, including objective knee indicators (KSS_Objective_), symptoms (KSS_Symptoms_), patient satisfaction (KSS_Satisfaction_) and expectations (KSS_Expectation_) and functional activities (KSS_Function_) [40]. KSS_Satisfaction_ is calculated based on five single items, with higher scores indicating a higher level of satisfaction (range: 0–40). Dissatisfaction was defined as KSS_Satisfaction_ ≤ 20 points. Furthermore, the Knee injury and Osteoarthritis Outcome Score (KOOS) [43] was determined at the same time points to assess the outcome regarding pain, symptoms, activities of daily living (ADL), functioning in sport and recreation (Sport/Rec) and knee‐related quality of life (QoL). Each scale was transformed into percentage points, with 0% representing the worst and 100% the best condition. Furthermore, pain intensity and psychological distress were assessed. Pain intensity within the last week was quantified using a VAS, measured on a 10 cm line ranging from ‘no pain’ (0) to ‘worst pain’ (10). Psychological distress, encompassing anxiety and depression, was assessed using the Patient Health Questionnaire‐4 screening [32]. Additionally, demographic variables such as social class, sex and age were investigated. Further examined variables were polyethylene insert thickness in millimetres, BMI, Kellgren–Lawrence (KL) osteoarthritis severity grade [24], anatomic tibiofemoral angle, hip–knee–ankle (HKA) angle (both to classify varus or valgus deformities) and preoperative range of motion (ROM).

### Sample size calculation

Sample size calculation was performed using G*Power software v. 3.1.9.2 (Heinrich‐Heine‐Universität Düsseldorf). With an alpha risk of 0.05, a statistical power of 0.8 and an effect size of *f*² = 0.09, a total sample size of at least 175 patients was required to calculate a multiple binary logistic regression with eight independent predictors of dissatisfaction. This effect size indicates a small to medium effect, capable of detecting significant small beta weights in multiple regression models. With a sample size of 236 patients at the time of analysis, the number of cases was adequate to achieve sufficient statistical power.

### Ethical approval and informed consent

The study was approved by the local ethics committee of the Medical Association of Hamburg (ID number: PV7275) and adhered to the principles of the Declaration of Helsinki. Before their participation, all patients provided written informed consent. Each patient was informed of the study's purpose, procedures, potential risks, benefits and confidentiality measures. The study adheres to the Transparent Reporting of a Multivariable Prediction Model for Individual Prognosis or Diagnosis checklist, ensuring transparent reporting of the development of the predictive model (Supporting Information S1: Table S1) [12].

### Statistical analysis

Continuous variables are expressed as mean ± standard deviation, while categorical variables are expressed as number and percentage. To compare satisfied and dissatisfied patients in terms of continuous variables, the Student's *t* test for independent samples was used for normally distributed data. The Mann–Whitney *U* test was used with nonnormally distributed data and *χ* ^2^ or Fisher's exact tests were applied for categorical variables. Repeated measures analysis of variance (ANOVA) was applied to determine significant differences between measurement points and patients. Effect sizes were expressed in terms of partial eta squared (*η* ^2^) for longitudinal analyses (0.01 ≙ small, 0.06 ≙ medium, 0.14 ≙ large effect size) or Cohen's *d* for group comparisons at one measurement point (0.2 ≙ small, 0.5 ≙ medium, 0.8 ≙ large effect size). All analyses were performed using SPSS v. 29 (IBM) for Windows. Statistical significance was set to a two‐tailed *p* value of 0.05.

The primary outcome was evaluated using single and multiple logistic regression analyses to identify significant predictors for patients' dissatisfaction 1 year after TKA. The study cohort was grouped into satisfied (KSS_Satisfaction_ > 20) and dissatisfied (KSS_Satisfaction_ ≤ 20) patients [36, 41]. As the first step in developing a clinical risk score for dissatisfaction, single logistic regressions were performed to determine whether age, sex, BMI, KL grade, ROM, varus or valgus deformity, insert thickness, VAS_Pain_, the KOOS scales, the KSS patient expectation scale (KSS_Expecation_) or psychological distress predict dissatisfaction (KSS_Satisfaction_ ≤ 20). For further analysis, optimal threshold values were determined for significant continuous predictors by means of the Youden J statistic and receiver operating curves (ROC) to discriminate between satisfied and unsatisfied patients [18]. In the second step, a multiple binary logistic regression was performed using categorised variables to predict dissatisfaction (KSS_Satisfaction_ ≤ 20). Finally, a weighted risk score was developed based on the β‐regression weights. ROC analyses and the negative and positive predictive values were calculated. Variables that improved the overall predictive value of the ROC model, despite failing to reach statistical significance in the multiple regression analysis, were included in the final risk score.

## RESULTS

### Demographic and disease characteristics of the study cohort

Of the 236 included patients, most patients were female (59.7%). The mean age was 68.8 ± 9.9 years and the mean BMI was 30.6 ± 5.9 kg/m^2^. Approximately half of the patients (51.3%) were diagnosed with a KL grade 4 and had a comorbidity Charnley class A (53.0%). In 63.0% of patients, the HKA angle indicated a varus deformity (<−3°). The mean preoperative VAS_Pain_ was 6.7 ± 2.1 and the mean preoperative ROM amounted 105.3 ± 14.9°. Furthermore, patients demonstrated a KSS_Objective_ of 46.6 ± 20.3 and a KSS_Function_ of 36.6 ± 16.8 preoperatively. Overall, 6.8% of the patients experienced severe and 10.2% moderate psychological distress (Table 1).

**Table 1:** Demographic and disease‐specific characteristics of the overall study cohort and differentiation between satisfied and dissatisfied patients 1 year after total knee arthroplasty.

	Overall (*n* = 236)	Satisfied patients (*n* = 199)	Dissatisfied patients (*n* = 37)	*p* Value
Age in years [mean (SD)]	68.8 (9.9)	69.5 (9.7)	65.4 (10.1)	**0.023**
Sex (*n*, %)				
Male	95, 40.3%	85, 42.7%	10, 27.0%	0.100
Female	141, 59.7%	114, 57.3%	27, 73.0%	
Body mass index [mean (SD)]	30.6 (5.9)	30.1 (5.7)	33.0 (6.2)	**0.007**
Sociological Class Index (*n*, %)				
Lower class	82, 34.7%	73, 36.7%	9, 24.3%	
Middle class	87, 36.9%	73, 37.7%	14, 7.8%	0.258
Upper class	62, 26.3%	49, 24.6%	13, 35.1%	
Missing	5, 2.1%	4, 2.0%	1, 2.7%	
Occupation (*n*, %)				
Full‐time	43, 18.2%	31, 15.6%	12, 32.4%	
Part‐time	20, 8.5%	16, 8.0%	4, 10.8%	
Unemployed	16, 6.8%	13, 6.5%	3, 8.1%	0.058
Retired	151, 64.0%	134, 67.3%	17, 45.9%	
Missing	6, 2.5%	5, 2.5%	1, 2.7%	
PHQ‐4 depression/anxiety at baseline (*n*, %)				
No symptoms	196, 83.1%	169, 84.9%	27, 73.0%	
Yellow flag (≥6 points)	24, 10.2%	19, 9.5%	5, 13.5%	0.138
Red flag (≥9 points)	16, 6.8%	11, 5.5%	5, 13.5%	
Charnley classification (*n*, %)				
A	125, 53.0%	108, 54.3%	17, 45.9%	
B	41, 17.4%	32, 16.1%	9, 24.3%	0.443
C	70, 29.7%	59, 29.6%	11, 29.7%	
Previous knee surgery (*n*, %)				
Yes	47, 19.9%	41, 20.6%	6, 16.2%	0. 657
No	189, 80.1%	158, 79.4%	31, 83.8%	
Range of motion (°) at baseline [mean (SD)]	105.3 (14.9)	105.9 (14.8)	102.2 (15.6)	0.162
VAS at baseline [mean (SD)]	6.7 (2.1)	6.7 (2.2)	7.1 (1.8)	0.306
KSS_Objective_ at baseline [mean (SD)]	46.6 (20.3)	46.7 (20.1)	46.2 (21.4)	0.903
KSS_Function_ at baseline [mean (SD)]	36.6 (16.8)	37.1 (17.2)	34.1 (14.6)	0.346
Kellgren–Lawrence classification (*n*, %)				
Grade 2	10, 4.3%	7, 3.5%	3, 8.1%	
Grade 3	105, 44.5%	91, 45.7%	14, 37.8%	0.358
Grade 4	121, 51.3%	101, 50.8%	20, 54.1%	
Anatomic tibiofemoral angle (*n*, %)				
Varus (<5°)	153, 64.4%	127, 63.8%	25, 67.6%	
Valgus (>8°)	55, 23.3%	46, 23.1%	9, 24.3%	0.701
Normal (5–8°)	29, 12.3%	26, 13.1%	3, 8.1%	
Hip–knee–ankle angle (*n*, %)				
Varus (<−3°)	149, 63.0%	126, 63.1%	23, 62.2%	
Valgus (>+3°)	45, 19.1%	38, 19.2%	7, 18.9%	0.984
Normal (−3 to +3°)	42, 17.9%	35, 17.7%	7, 18.9%	

*Note*: Satisfaction is defined as a KSS satisfaction scale >20 1 year after TKA; dissatisfaction is defined as a KSS satisfaction scale ≤ 20 one year after TKA. Significant differences are displayed in bold.

Abbreviations: KSS, Knee Society Score; PHQ, Patient Health Questionnaire; SD, standard deviation; TKA, total knee arthroplasty; VAS, visual analogue scale.

### Satisfaction and PROMs after TKA

**Figure 2:**
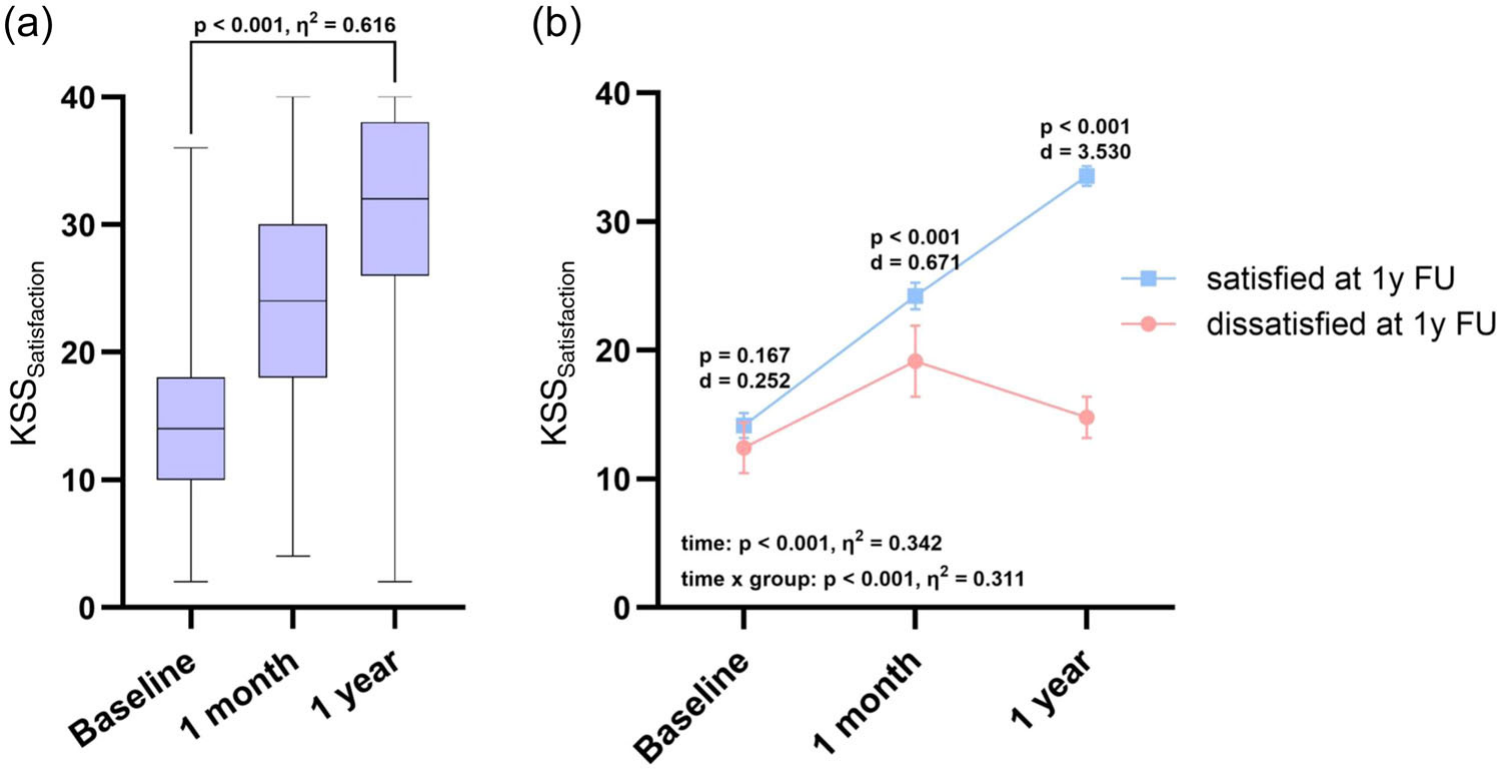
Improvement of patients’ satisfaction after total knee arthroplasty (TKA). (a) Improvement in patient satisfaction up to 1 year after TKA for the entire study cohort, assessed by the Knee Society Score (KSS) satisfaction scale. Box‐and‐Whisker‐plots, representing median, interquartile range and minimum and maximum, are displayed. (b) Course of patient satisfaction, divided into satisfied patients (KSS_Satisfaction_ > 20) and dissatisfied patients at 1 year (KSS_Satisfaction_ ≤ 20), analysed using repeated measures analysis of variance. Mean ± standard deviation is displayed.

**Figure 3:**
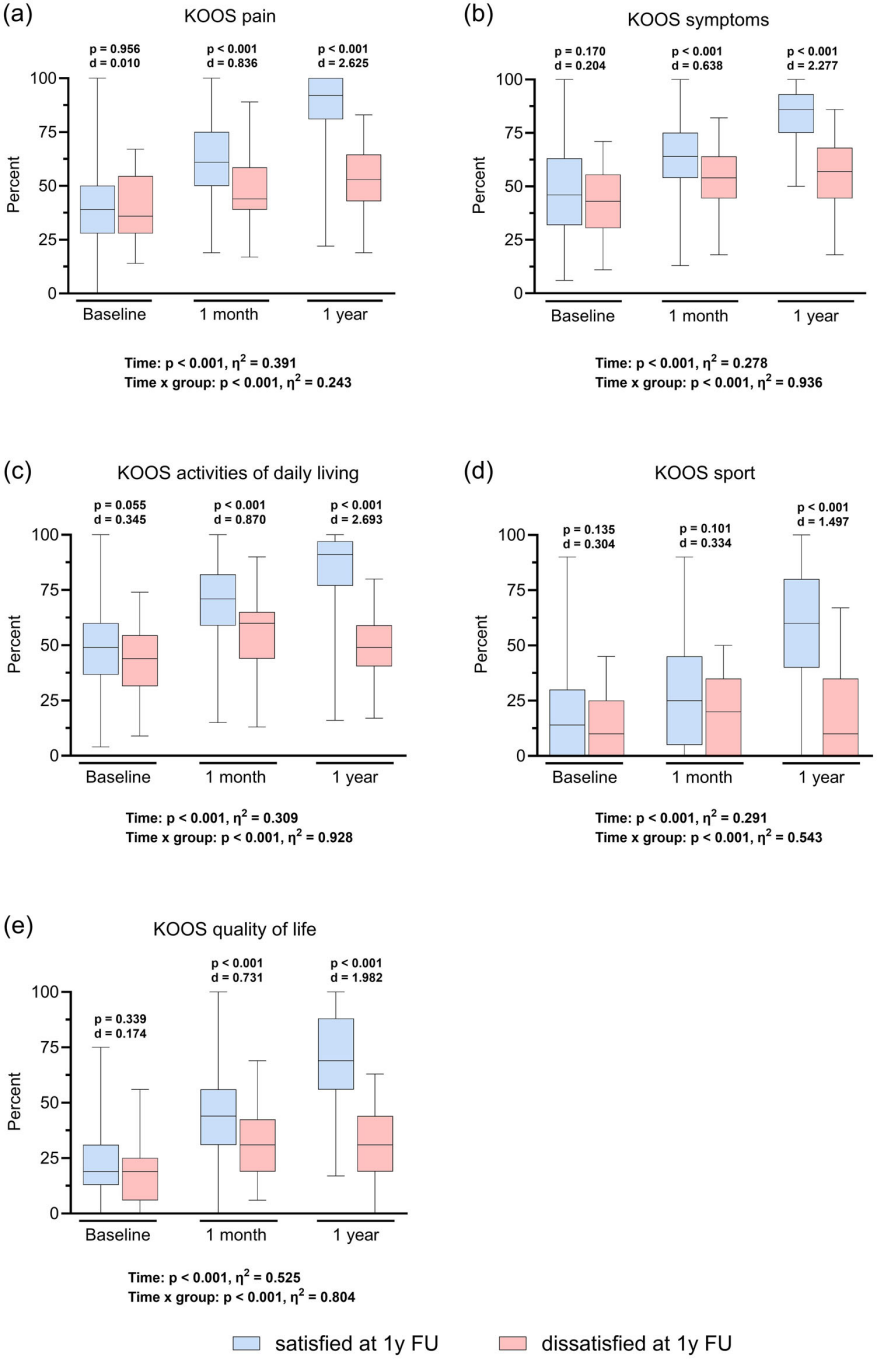
Changes in clinical outcome parameters comparing satisfied and dissatisfied patients. Repeated measures analysis of variance analyses were conducted to determine the improvement in patient‐reported outcome parameters measured by the Knee injury and Osteoarthritis Outcome Score (KOOS) at baseline, 1 month and 1 year after total knee arthroplasty (TKA) stratified by satisfied patients (Knee Society Score [KSS]_Satisfaction_ > 20) and dissatisfied patients (KSS_Satisfaction_ ≤ 20) at 1 year follow‐up (FU). At each measurement point, Box‐and‐Whisker‐plots, representing median, interquartile range and minimum and maximum, are displayed for the KOOS (a) pain scale, (b) symptoms scale, (c) activities of daily living scale, (d) functioning in sport and recreation scale and (e) quality of life scale.

Overall, KSS_Satisfaction_ increased significantly (*p* < 0.001) from preoperatively (13.9 ± 6.8) to 1 year postoperatively (30.6 ± 8.7) (Figure 2a). One year after TKA, 16% of the patients were dissatisfied, scoring a KSS_Satisfaction_ ≤ 20 points. There was a significant group × time interaction regarding the changes in satisfaction (*p* < 0.001). Hence, the longitudinal analysis demonstrated no significant difference between the dissatisfied and satisfied patients preoperatively (Δ1.8; *p* = 0.167). Significant differences between both groups emerged after 1 month (Δ4.9; *p* < 0.001) and 1 year postoperatively (Δ18.6; *p* < 0.001) (Figure 2b). Dissatisfied patients were significantly younger (*p* = 0.023) and had a higher BMI (*p* = 0.007). No further differences between satisfied and dissatisfied patients were observed with regard to other demographic or clinical variables (Table 1). None of the KOOS scales demonstrated significant differences preoperatively. However, within 1 month, dissatisfied patients already reported significantly more pain (KOOS_Pain_: Δ14.3; *p* < 0.001), a higher symptom burden (KOOS_Symptoms_: Δ10.0; *p* < 0.001), a limited ability to perform ADL (KOOS_ADL_: Δ14.2; *p* < 0.001) and reduced QoL (KOOS_QoL_: Δ13.7; *p* < 0.001). These effects increased over time. At 1 year follow‐up, there was also a significant difference regarding the KOOS_Sport/Rec_ (Δ40.6; *p* < 0.001). Overall, there was significant improvement in all KOOS scales up to 1 year after TKA (*p* < 0.001), with significant time × group effects in all scales (Figure 3a–e).

### Development and validation of a clinical risk score for dissatisfaction

To identify patients at risk of dissatisfaction, the development of an early decision aid was intended. In the first step, logistic regression analyses were computed to identify single variables that predict a KSS_Satisfaction_ ≤ 20 points. Age, BMI, VAS_Pain_, KOOS_Pain_, KOOS_Symptoms_, KSS_Objective_, KSS_Function_ and KSS_Expectation_ (all at 1 month postoperatively) were identified as significant single predictors. Besides age and BMI, no other preoperatively available variables were found to be significant predictive factors. Predictors that failed to meet clinical considerations or regression criteria (*r*_Pearson_ > 0.7) were excluded (Supporting Information S1: Table S2). In the next step, Youden's *J* statistic was used to calculate the optimal threshold for all interval‐scaled variables. The subsequent multiple binary logistic regression analysis with backwards elimination was statistically significant (*p* < 0.001) and accounted for 32.5% of the variance (Nagelkerkes *R*²) (Table 2, Supporting Information S1: Table S3). In the last step of score development, the significant variables age, KOOS_Pain_ and KSS_Expectation_ identified in the multiple regression analysis were integrated into a weighted risk score. Though not statistically significant in the multiple regression, BMI and the KSS_Function_ scale were additionally integrated into the score, as they enhanced the overall model performance examined by ROC analyses (Supporting Information S1: Figure S1).

**Table 2:** Multiple logistic regression for independent dichotomous predictors of KSS_Satisfaction_ ≤ 20 at 1 year after total knee arthroplasty.

								95% CI	
	Predictor	*β*	SE *β*	Wald's *χ* ^2^	*df*	*p* Value	OR	Lower	Upper
Last elimination step	KSS_Expectation_	1.065	0.474	5.053	1	0.025	2.902	1.146	7.348
	KOOS_Pain_	1.907	0.462	17.067	1	<0.001	6.730	2.724	16.629
	Age	1.198	0.468	6.562	1	0.010	3.314	1.325	8.291
	Constant	−3.195	0.442	52.269	1	<0.001	0.041	‐	‐

	**Test**			***χ*** ^**2**^	***df***	*p* Value			
	Overall model evaluation			40.331	3	<0.001			
	Omnibus‐test								
	Goodness‐of‐fit test			0.614	4	0.962			
	Hosmer–Lemeshow test								

*Note*: Result from the multiple binary logistic regression model with ‘backwards elimination’ method. Cox and Snell *R*² = 0.196; Nagelkerkes *R*² = 0.325. The full regression model with all elimination steps is displayed in Supporting Information S1: Table S3.

Abbreviations: CI, confidence interval; *df*, degrees of freedom; KOOS, Knee injury and Osteoarthritis Outcome Score; KSS, Knee Society Score; OR, odds ratio; SE, standard error.

**Figure 4:**
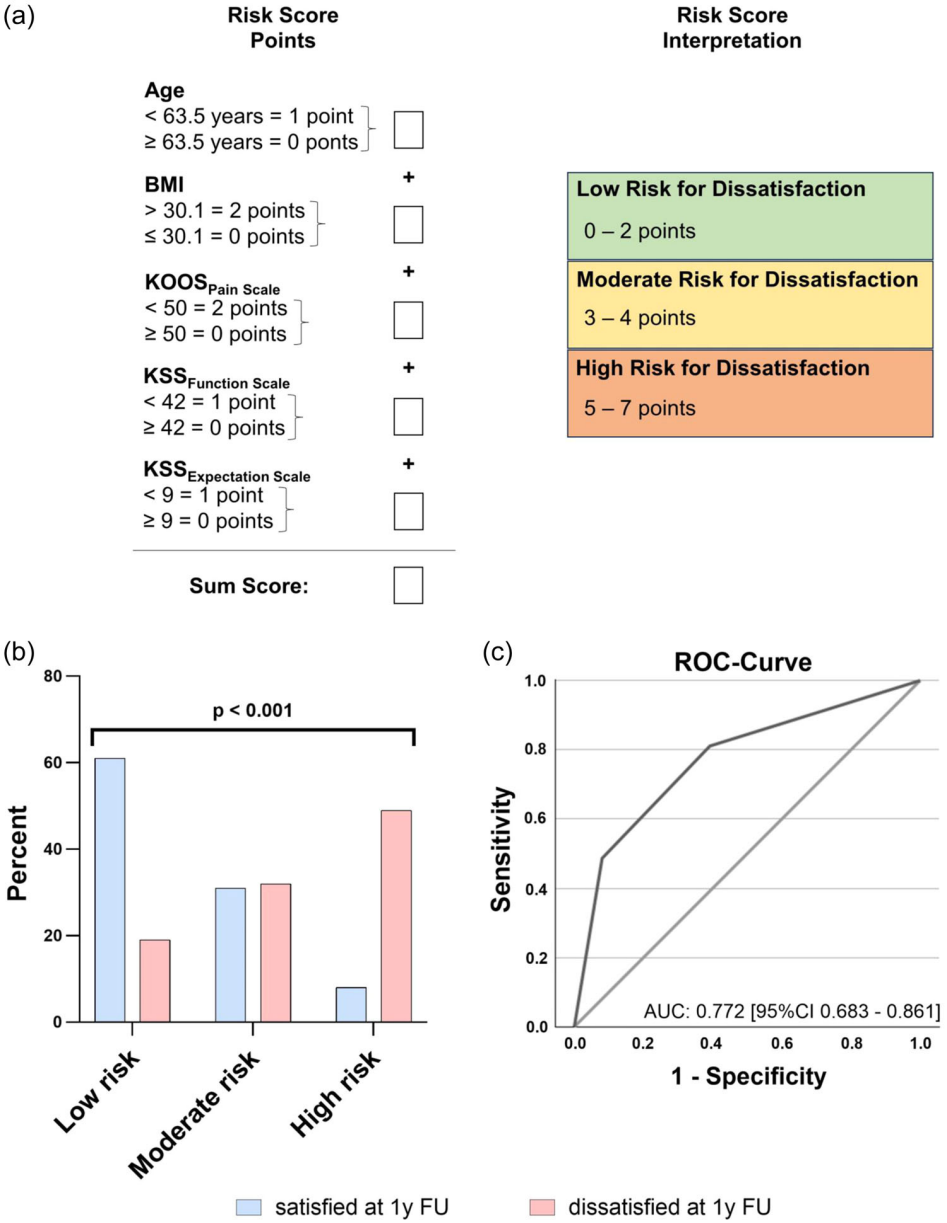
Predictive value of the developed risk score for patient dissatisfaction 1 year after total knee arthroplasty. (a) Overview of the newly developed dissatisfaction prediction score with instructions for calculation and interpretation. (b) Likelihood of satisfaction and dissatisfaction based on the calculated risk category (low, moderate and high risk). The risk categories significantly differentiate between satisfied (Knee Society Score [KSS]_Satisfaction_ > 20) and dissatisfied (KSS_Satisfaction_ ≤ 20) patients (*χ* ^2^ test). (c) Receiver operating characteristic (ROC)‐curve yielding an area under the curve (AUC) of 0.772, indicating a robust ability of the risk score to predict patient dissatisfaction. BMI, body mass index; CI, confidence interval; FU, follow‐up.

The developed risk score consisted of a maximum of seven points (Figure 4a). The included variables demonstrated that the likelihood of postoperative dissatisfaction 1 year postoperative was 3.0, 4.0, 7.4, 4.3 and 2.8 times higher for patients younger than 63.5 years of age, with a BMI over 30.1 kg/m², KOOS_Pain_ scale less than 50%, KSS_Function_ under 42 points and KSS_Expectation_ lower than nine points, respectively. The score achieved a good predictive value for dissatisfaction, indicating a robust discriminative ability (AUC: 0.792 [95% confidence interval, CI: 0.700–0.884], *p* < 0.001). This model achieved the highest AUC of all scoring options tested. For practical implementation, the score was divided into three risk categories: Low risk (0–2 points), moderate risk (3–4 points) and high risk (5–7 points) for dissatisfaction (Figure 4b). This categorisation demonstrated good precision to discriminate between these three risk groups (Figure 4c; AUC: 0.772; [95% CI: 0.683–0.861]; *p* < 0.001). Comparing the low‐ and high‐risk group, the score achieved a sensitivity of 0.720 [95% CI: 0.524–0.857], a specificity of 0.883 [95% CI: 0.818–0.927], a positive predictive value of 0.529 [95% CI: 0.367–0.686] as well as an excellent negative predictive value of 0.945 [95% CI: 0.891–0.973]. A high‐risk classification was associated with an odds ratio of 9.7 [95% CI: 4.5–20.9] for experiencing dissatisfaction 1 year after TKA.

## DISCUSSION

The most important finding of the present study was that dissatisfaction 1 year after TKA can be predicted by a newly developed risk score. This score comprises the preoperatively available patient‐specific variables age and BMI, as well as the 1‐month postoperative variables pain (KOOS_Pain_), functionality (KSS_Function_) and unmet expectations (KSS_Expectation_). Our model identified patients at risk of dissatisfaction as early as 1 month after TKA, allowing early initiation of preventive measures. Given the high prevalence of long‐term dissatisfaction in TKA patients, the prediction of dissatisfaction has high clinical relevance in daily orthopaedic practice.

Currently, there is a lack of standardised follow‐up care for patients after TKA, as each hospital follows its own protocols. Although physiotherapy can significantly improve the functional capacity [31], the length and intensity of rehabilitation vary depending on the clinical setting and geographic region [16, 27]. Because some patients do not have structured follow‐up after surgery, there is a risk that dissatisfied patients will go unnoticed. Therefore, a risk score can identify dissatisfied patients at an early stage, thus enabling clinical re‐evaluation and targeted interventions such as adjusting physiotherapy, optimising pain management or psychosocial support. Our clinical risk score included five factors associated with patient dissatisfaction: younger patient age, higher BMI, more severe pain, lower subjective functionality and unmet expectations 1 month after TKA. The identified factors are generally in line with the results of previous studies [9, 13, 22, 34]. Unlike other studies, we were unable to identify any additional preoperative predictors within our study population. Specifically, none of the other known preoperative influencing factors, for example, sex [10], PROMs [17], osteoarthritis severity [19], KSS [28] or psychological distress [41], were significant predictors in our study. In this context, it is important that preoperative factors alone were also not able to explain postoperative satisfaction in a previous joint registry study [3]. Notably, when examining factors that influence satisfaction, satisfaction and predictor variables are often examined at the same time point. Consistent with this, our regression analyses suggested that the most influential factors on 1‐year satisfaction were not preoperative factors, but variables measured at the same time point as the satisfaction assessment. Characterised by higher beta weights and higher variance, these variables had a more pronounced impact (Supporting Information S1: Table S2). Importantly, however, our results indicate that predicting dissatisfaction was feasible as early as 1 month after TKA.

In the group comparisons between patients categorised as satisfied and dissatisfied 1 year after TKA, differences in patient outcomes were already observed after 1 month. Specifically, there was a significant difference in four of five scales of the KOOS between satisfied and dissatisfied patients. This effect persisted and even intensified up to 1 year after surgery, resulting in large effect sizes. We further observed that although the outcomes improved over time in both groups, the improvement in the dissatisfied patients was significantly less. Based on the early differentiation between satisfied and dissatisfied patients, the regression model was able to identify significant predictors at an early stage to incorporate them into a risk score. In addition to age and BMI, our score consists of the patient‐reported KOOS pain scale, the KSS functional scale (KSS_Function_) and the KSS expectation scale (KSS_Expectation_) 1 month postoperatively. The KOOS pain scale comprises nine items and exhibits no floor effects. It has strong internal consistency, construct validity and responsiveness, as well as good test–retest reliability [11]. The KSS_Function_ is longer and consists of 19 items to assess patient‐specific activities, ADL, sports and recreational activities [40]. The score is also a reliable and internally consistent instrument with good construct validity and responsiveness [14]. The KSS_Expectation_ consists of three items, whereby low scores represent high expectations. In accordance with the results of a previous study [44], we observed no correlation between patients' preoperative expectations and satisfaction at 1 year postoperatively (*r* = 0.065; *p* = 0.320). However, we were able to identify a significant correlation between patient expectations after 1 month and satisfaction 1 year postoperatively (*r* = 0.230; *p* < 0.001).

All items of the developed risk score can be presented to patients for self‐completion, eliminating the need for further physical examination. In contrast to the results of Van Onsem et al. [41], which developed a linear score to predict satisfaction at 3 months, our research focused on dissatisfaction 1 year after TKA. The risk score allows classification into three risk groups (low, moderate and high) and has a robust ability to discriminate between satisfied and dissatisfied patients. Therefore, our dissatisfaction risk score can serve as a screening tool during postoperative assessments to identify patients at higher risk of dissatisfaction in the longer term.

This study has clear strengths: It is the first study to develop a clinical screening tool focussing on dissatisfaction 1 year after TKA. Only established and standardised clinical variables and PROMs were used, which ensures the validity of the data and conclusions. Acknowledging the prevalent challenges posed by the heterogeneous methodologies and low levels of evidence found in numerous studies on patient satisfaction after TKA [23], we chose to utilise the prevalidated KSS scale to assess satisfaction. In determining the cut‐offs that differentiate satisfaction from dissatisfaction, our methodology was guided by the existing literature [36, 41]. The KSS_Satisfaction_ scale offers a more comprehensive and multidimensional evaluation of patient satisfaction following TKA. This approach may be superior to the single‐item Likert scales commonly used in previous studies to assess satisfaction. A satisfaction scale includes multiple items that assess various aspects of satisfaction, such as pain relief, functional improvement and overall well‐being. This approach provides a more comprehensive understanding of patient satisfaction, encompassing the intricacy of the patient experience beyond a basic overall rating.

Our study also has several limitations. This was an observational study as part of a single‐centre patient registry. Because of the observational study design, it was not possible to control for all confounding factors that could affect the results. There is also a risk of data inconsistencies, missing information and bias introduced during data collection. To address these issues, a homogeneous cohort of patients was studied by applying specific inclusion and exclusion criteria. Only patients undergoing TKA for primary osteoarthritis, in whom one specific implant was used and in whom no major complications requiring revision surgery occurred (e.g., infection, fracture, loosening) were included. This approach was chosen because complications requiring hospital readmission are a key factor influencing satisfaction [4, 21] and would have a strong impact on the results. Another limitation of our study is the restricted focus on patient satisfaction at a single time point 1 year postoperatively. Since satisfaction following TKA is a multifaceted construct that may evolve over time, longer follow‐up periods could provide additional insights into the long‐term effects of surgery and postoperative care. A further limitation of our study is that the risk score was developed using a specific patient cohort. It is, therefore, possible that the scoring system is too focused on the characteristics of this cohort, making it difficult to generalise the risk score to, for example, other populations or implant systems. Nevertheless, within the cohort studied, the analyses appeared to be conclusive and had high internal validity, as a sufficient number of patients met the requirements of the sample size calculation. Despite the inclusion of all the variables originally examined in the standardised framework of this study, there may be other factors that influence satisfaction (e.g., self‐efficacy or trait anxiety). Our statistical model explained 32.5% of the variance, suggesting that unmeasured variables may also play a role. Nevertheless, the variance explained in our multiple regression is similar to that reported in a previous study [41].

## CONCLUSION

In conclusion, dissatisfaction 1 year after TKA can be predicted by a developed risk score based on age, BMI, pain, subjective functionality and unmet expectations (assessed 1 month after TKA). Early identification of patients who are likely to be dissatisfied allows for targeted interventions, additional support and optimised resources.

## AUTHOR CONTRIBUTIONS

**André Strahl**: Conceptualisation; data curation; formal analysis; investigation; methodology; project administration; visualisation; writing—original draft; writing—review and editing. **Maximilian M. Delsmann**: Investigation; writing—review and editing. **Alexander Simon**: Formal analysis; validation; writing—review and editing. **Christian Ries**: Investigation; resources; writing—review and editing. **Tim Rolvien**: Conceptualisation; investigation, supervision; writing—original draft; writing—review and editing. **Frank Timo Beil**: Investigation; resources; supervision; writing—review and editing.

## CONFLICT OF INTEREST STATEMENT

The authors declare no conflict of interest.

## ETHICS STATEMENT

The study conformed to the principles of the Declaration of Helsinki and was approved by the local research ethics committee of the Medical Association Hamburg (Date: 23.06.2020/No: PV7275). Informed written consent was obtained from all individual participants included in the study.

## Supporting information

Supporting information.
