# Supplementary material for: A clinical risk score enables early prediction of dissatisfaction 1 year after total knee arthroplasty
Source: Knee Surg Sports Traumatol Arthrosc. 2024 May 26;33(1):252–64. doi: 10.1002/ksa.12277 (PMC11716356; doi:10.1002/ksa.12277)
Supplement: Supplementary file 1 — Supporting information. [file KSA-33-252-s001.docx]

| **Section/Topic** | **Item** | **Checklist Item** | **Page  (based on the uploaded Word Document)** |
| --- | --- | --- | --- |
| **Title and abstract** | | | |
| Title | 1 | Identify the study as developing and/or validating a multivariable prediction model, the target population, and the outcome to be predicted. | 1 |
| Abstract | 2 | Provide a summary of objectives, study design, setting, participants, sample size, predictors, outcome, statistical analysis, results, and conclusions. | 1 |
| **Introduction** | | | |
| Background and objectives | 3a | Explain the medical context (including whether diagnostic or prognostic) and rationale for developing or validating the multivariable prediction model, including references to existing models. | 2 |
|  | 3b | Specify the objectives, including whether the study describes the development or validation of the model or both. | 2 |
| **Methods** | | | |
| Source of data | 4a | Describe the study design or source of data (e.g., randomized trial, cohort, or registry data), separately for the development and validation data sets, if applicable. | 3 |
|  | 4b | Specify the key study dates, including start of accrual; end of accrual; and, if applicable, end of follow-up. | 3 |
| Participants | 5a | Specify key elements of the study setting (e.g., primary care, secondary care, general population) including number and location of centres. | 3 |
|  | 5b | Describe eligibility criteria for participants. | 3, Figure 1 |
|  | 5c | Give details of treatments received, if relevant. | 3 |
| Outcome | 6a | Clearly define the outcome that is predicted by the prediction model, including how and when assessed. | 3 |
|  | 6b | Report any actions to blind assessment of the outcome to be predicted. | no blinding carried out |
| Predictors | 7a | Clearly define all predictors used in developing or validating the multivariable prediction model, including how and when they were measured. | 4-5, Table 2, Supl. Tabl. S2, Supl. Tabl. S3 |
|  | 7b | Report any actions to blind assessment of predictors for the outcome and other predictors. | no blinding carried out |
| Sample size | 8 | Explain how the study size was arrived at. | 4-5, All patients meeting the eligibility criteria in the retrospective design |
| Missing data | 9 | Describe how missing data were handled (e.g., complete-case analysis, single imputation, multiple imputation) with details of any imputation method. | 3 complete-case analysis |
| Statistical analysis methods | 10a | Describe how predictors were handled in the analyses. | Categorisation, 5, 6-7 |
|  | 10b | Specify type of model, all model-building procedures (including any predictor selection), and method for internal validation. | 5-6 |
|  | 10d | Specify all measures used to assess model performance and, if relevant, to compare multiple models. | 5-7 |
| Risk groups | 11 | Provide details on how risk groups were created, if done. | 6, Figure 4 |
| **Results** | | | |
| Participants | 13a | Describe the flow of participants through the study, including the number of participants with and without the outcome and, if applicable, a summary of the follow-up time. A diagram may be helpful. | 3, Figure 1 |
|  | 13b | Describe the characteristics of the participants (basic demographics, clinical features, available predictors), including the number of participants with missing data for predictors and outcome. | 5, Table 1 |
| Model development | 14a | Specify the number of participants and outcome events in each analysis. | Table 1, complete-case analysis |
|  | 14b | If done, report the unadjusted association between each candidate predictor and outcome. | Single regressions, Suppl. Table S2 |
| Model specification | 15a | Present the full prediction model to allow predictions for individuals (i.e., all regression coefficients, and model intercept or baseline survival at a given time point). | Table 2, Suppl. Table S3 |
|  | 15b | Explain how to the use the prediction model. | 6, Fig. 4 |
| Model performance | 16 | Report performance measures (with CIs) for the prediction model. | 6-7 |

| **Discussion** | | | |
| --- | --- | --- | --- |
| Limitations | 18 | Discuss any limitations of the study (such as nonrepresentative sample, few events per predictor, missing data). | 9 |
| Interpretation | 19b | Give an overall interpretation of the results, considering objectives, limitations, and results from similar studies, and other relevant evidence. | 7-9 |
| Implications | 20 | Discuss the potential clinical use of the model and implications for future research. | 7 |
| **Other information** | | | |
| Supplementary information | 21 | Provide information about the availability of supplementary resources, such as study protocol, Web calculator, and data sets. | not applicable |
| Funding | 22 | Give the source of funding and the role of the funders for the present study. | No funding |

**Supplementary Table S2: Single logistic regression analyses to predict the satisfaction scale of the Knee Society Score at one year after TKA**

|  |  |  |  |  |  |  | **95% CI** | |  |
| --- | --- | --- | --- | --- | --- | --- | --- | --- | --- |
| **Predictor** | **β** | **SE β** | **Wald’s χ^2^** | ***df*** | ***p-*value** | **OR** | **Lower** | **Upper** | **In- or exclusion** |
| **Age** | **-0.040** | **0.028** | **5.030** | **1** | **0.025** | **0.961** | **0.928** | **0.995** | **Inclusion** |
| Sex (female) | 0.700 | 0.397 | 3.107 | 1 | 0.078 | 2.013 | 0.925 | 4.383 | Exclusion, not significant |
| **BMI** | **0.075** | **0.029** | **6.853** | **1** | **<0.001** | **1.078** | **1.019** | **1.140** | **Inclusion** |
| Social class index | 0.125 | 0.105 | 1.425 | 1 | 0.233 | 1.134 | 0.923 | 1.393 | Exclusion, not significant |
| Charnley classification | 0.052 | 0.088 | 0.342 | 1 | 0.559 | 1.053 | 0.886 | 1.252 | Exclusion, not significant |
| Previous knee surgery | -0.293 | 0.479 | 0.374 | 1 | 0.541 | 0.746 | 0.292 | 1.908 | Exclusion, not significant |
| KL-score | -0.039 | 0.309 | 0.016 | 1 | 0.901 | 0.962 | 0.526 | 1.762 | Exclusion, not significant |
| Insert height | -0.032 | 0.105 | 0.093 | 1 | 0.761 | 0.969 | 0.789 | 1.190 | Exclusion, not significant |
| ROM | -0.016 | 0.012 | 1.947 | 1 | 0.163 | 0.984 | 0.962 | 1.007 | Exclusion, not significant |
| aTFA - varus knee axis | 0.188 | 0.381 | 0.244 | 1 | 0.621 | 1.207 | 0.572 | 2.546 | Exclusion, not significant |
| aTFA - Valgus knee axis | 0.067 | 0.418 | 0.025 | 1 | 0.873 | 1.069 | 0.471 | 2.428 | Exclusion, not significant |
| HKA angle - varus knee axis | -0.063 | 0.370 | 0.029 | 1 | 0.864 | 0.939 | 0.455 | 1.938 | Exclusion, not significant |
| HKA angle - Valgus knee axis | -0.018 | 0.457 | 0.002 | 1 | 0.969 | 0.982 | 0.401 | 2.406 | Exclusion, not significant |
| VAS pain t0 | 0.092 | 0.090 | 1.051 | 1 | 0.305 | 1.097 | 0.919 | 1.308 | Exclusion, not significant |
| **VAS pain t1** | **0.314** | **0.094** | **11.026** | **1** | **<0.001** | **1.368** | **1.137** | **1.646** | **Inclusion** |
| VAS pain t2 | 1.144 | 0.179 | 40.827 | 1 | <0.001 | 3.138 | 2.210 | 4.457 | Exclusion,  no early predictor |
| KOOS_Pain_ t0 | -0.001 | 0.011 | 0.003 | 1 | 0.956 | 0.999 | 0.978 | 1.021 | Exclusion, not significant |
| **KOOS_Pain_ t1** | **-0.055** | **0.013** | **17.698** | **1** | **<0.001** | **0.947** | **0.923** | **0.971** | **Inclusion** |
| KOOS_Pain_ t2 | -0.133 | 0.020 | 44.977 | 1 | <0.001 | 0.876 | 0.842 | 0.910 | Exclusion,  no early predictor |
| KOOS_Symptoms_ t0 | -0.010 | 0.009 | 1.255 | 1 | 0.263 | 0.990 | 0.972 | 1.008 | Exclusion, not significant |
| **KOOS_Symptoms_ t1** | **-0.038** | **0.011** | **11.445** | **1** | **<0.001** | **0.962** | **0.941** | **0.984** | **Inclusion** |
|  |  |  |  |  |  |  | **95% CI** | |  |
| **Predictor** | **β** | **SE β** | **Wald’s χ^2^** | ***df*** | ***p-*value** | **OR** | **Lower** | **Upper** | **In- or exclusion** |
| KOOS_Symptoms_ t2 | -0.150 | 0.023 | 41.310 | 1 | <0.001 | 0.861 | 0.823 | 0.901 | Exclusion,  no early predictor |
| KOOS_ADL_ t0 | -0.020 | 0.010 | 3.610 | 1 | 0.057 | 0.980 | 0.960 | 1.001 | Exclusion, not significant |
| KOOS_ADL_ t1 | -0.049 | 0.011 | 18.676 | 1 | <0.001 | 0.952 | 0.931 | 0.974 | Exclusion,  high correlation (>0.7) with KOOS pain t1 |
| KOOS_ADL_ t2 | -0.135 | 0.021 | 41.827 | 1 | <0.001 | 0.873 | 0.838 | 0.910 | Exclusion,  no early predictor |
| KOOS_Sport/Rec_ t0 | -0.019 | 0.011 | 2.824 | 1 | 0.093 | 0.981 | 0.959 | 1.003 | Exclusion, not significant |
| KOOS_Sport/Rec_ t1 | -0.017 | 0.009 | 3.205 | 1 | 0.073 | 0.983 | 0.065 | 1.002 | Exclusion, not significant |
| KOOS_Sport/Rec_ t2 | -0.062 | 0.010 | 35.946 | 1 | <0.001 | 0.940 | 0.922 | 0.959 | Exclusion,  no early predictor |
| KOOS_QoL_ t0 | -0.012 | 0.013 | 0.862 | 1 | 0.353 | 0.988 | 0.962 | 1.014 | Exclusion, not significant |
| KOOS_QoL_ t1 | -0.045 | 0.012 | 14.340 | 1 | <0.001 | 0.956 | 0.934 | 0.978 | Exclusion,  clinical justification, as improved functionality results in improved QoL |
| KOOS_QoL_ t2 | -0.116 | 0.019 | 35.605 | 1 | <0.001 | 0.891 | 0.858 | 0.925 | Exclusion,  no early predictor |
| KSS_Objective_ t0 | -0.001 | 0.010 | 0.015 | 1 | 0.903 | 0.999 | 0.980 | 1.018 | Exclusion, not significant |
| **KSS_Objective_ t1** | **-0.025** | **0.016** | **2.627** | **1** | **0.105** | **0.975** | **0.946** | **1.005** | **Inclusion,  clinical justification, as objective functionality may be relevant factor** |
| KSS_Objective_ t2 | -0.208 | 0.031 | 44.063 | 1 | <0.001 | 0.812 | 0.763 | 0.863 | Exclusion,  no early predictor |
| KSS_Subjective_ t0 | -0.011 | 0.011 | 0.893 | 1 | 0.345 | 0.989 | 0.967 | 1.012 | Exclusion, not significant |
| **KSS_Subjective_ t1** | **-0.040** | **0.013** | **9.201** | **1** | **0.002** | **0.960** | **0.936** | **0.986** | **Inclusion** |
| KSS_Subjective_ t2 | -0.083 | 0.013 | 37.897 | 1 | <0.001 | 0.921 | 0.897 | 0.945 | Exclusion,  no early predictor |
| KSS_Expectation_ t0 | -0.175 | 0.096 | 3.330 | 1 | 0.068 | 0.839 | 0.696 | 1.013 | Exclusion, not significant |

|  |  |  |  |  |  |  | **95% CI** | |  |
| --- | --- | --- | --- | --- | --- | --- | --- | --- | --- |
| **Predictor** | **β** | **SE β** | **Wald’s χ^2^** | ***df*** | ***p-*value** | **OR** | **Lower** | **Upper** | **In- or exclusion** |
| **KSS_Expectation_ t1** | **-0.206** | **0.074** | **7.630** | **1** | **0.006** | **0.814** | **0.704** | **0.942** | **Inclusion** |
| KSS_Expectation_ t2 | -0.739 | 0.114 | 42.275 | 1 | <0.001 | 0.478 | 0.382 | 0.597 | Exclusion,  no early predictor |
| Psychological distress: Red flag t0 | 1.031 | 0.574 | 3.223 | 1 | 0.073 | 2.803 | 0.910 | 8.637 | Exclusion, not significant |
| Psychological distress: Red flag t1 | 0.891 | 0.630 | 2.000 | 1 | 0.157 | 2.438 | 0.709 | 8.380 | Exclusion, not significant |
| Psychological distress: Red flag t2 | 2.049 | 0.787 | 6.782 | 1 | 0.009 | 7.758 | 1.660 | 36.254 | Exclusion,  no early predictor |

ADL = Activities of daily living; aTFA = Anatomic tibiofemoral angle; BMI = Body mass index; HKA angle = Hip-Knee-Ankle angle; KL = Kellgren-Lawrence; KOOS = Knee injury and Osteoarthritis Outcome Score; KSS = Knee Society Score; OR = Odds ratio; QoL = Quality of life; ROM = Range of motion; SE = Standard error; Sport/Rec = Function in sport and recreation; TKA = Total knee arthroplasty; t0 = Baseline measurement time point; t1 = Measurement time point one month after TKA; t2 = Measurement time point twelve months after TKA; VAS = Visual Analogue Scale

**Supplementary Table S3: Multiple logistic regression for independent dichotomous predictors of KSS_Satisfaction_ ≤20 at one year after TKA**

|  |  |  |  |  |  |  |  | **95% CI** | |
| --- | --- | --- | --- | --- | --- | --- | --- | --- | --- |
|  | **Predictor** | **β** | **SE β** | **Wald’s χ^2^** | ***df*** | ***p-*value** | **OR** | **Lower** | **Upper** |
| Step 1 | KSS_Objective_  KSS_Function_ KSS_Expectation_ KOOS_Pain_ KOOS_Symptoms_ VAS Pain Age BMI Constant | 0.111 0.502 0.867 1.173 0.963 0.560 0.744 0.650 -4.141 | 0.530 0.507 0.502 0.560 0.552 0.562 0.522 0.512 0.651 | 0.043 0.981 2.977 4.388 3.044 0.994 2.032 1.616 40.473 | 1 1  1 1 1 1 1 1 1 | 0.835 0.322 0.084 0.036 0.081 0.319 0.154 0.204 <0.001 | 1.117 1.652 2.380 3.231 2.621 1.751 2.105 1.916 0.016 | 0.395 0.612 0.889 1.078 0.888  0.582 0.756 0.703 --- | 3.156 4.463 6.371 9.683 7.735  5.270 5.857 5.223 --- |
| Step 2 | KSS_Function_ KSS_Expectation_ KOOS_Pain_ KOOS_Symptoms_  VAS Pain Age BMI Constant | 0.496 0.883 1.179 0.978 0.588 0.762 0.671 -4.123 | 0.505 0497 0.559 0.548 0.546 0.515 0.503 0.646 | 0.963 3.165 4.448 3.191 1.164 2.186 1.781 40.792 | 1 1 1 1 1 1  1 1 | 0.326 0.075 0.035 0.074 0.281 0.139 0.182 <0.001 | 1.641 2.419 3.251 2.660 1.801 2.143 1.956 0.016 | 0.610 0.914 1.087 0.909 0.618 0.780 0.730 --- | 4.416 6.402 9.722 7.783 5.247 5.884 5.237 --- |
| Step 3 | KSS_Expectation_ KOOS_Pain_ KOOS_Symptoms_ VAS Pain Age BMI Constant | 0.888 1.321 1.008 0.684 0.828 0.691 -4.056 | 0.494 0.538 0.540 0.535 0.504 0.500 0.633 | 3.230 6.028 3.490 1.635 2.699 1.912 40.990 | 1 1 1 1 1 1 1 | 0.072 0.014 0.062 0.201 0.100 0.167 <0.001 | 2.430 3.748 2.740 1.981 2.290 1.996 0.017 | 0.923 1.305 0.952 0.695 0.852 0.749 --- | 6.402 10.763 7.890 5.650 6.153 5.314 --- |
| Step 4 | KSS_Expectation_ KOOS_Pain_ KOOS_Symptoms_ Age BMI  Constant | 0.994 1.644 0.896 0.822 0.694  -3.890 | 0.484 0.479 0.527 0.503 0.496  0.603 | 4.223 11.786 2.891 2.673 1.961  41.647 | 1 1 1 1 1 1 | 0.040 <0.001 0.089 0.102 0.161  <0.001 | 2.703 5.173 2.450 2.276 2.002  0.020 | 1.047 2.024 0.872 0.849 0.758  --- | 6.978 13.221 6.883 6.098 5.289  --- |
| Step 5 | KSS_Expectation_ KOOS_Pain_ KOOS_Symptoms_ Age  Constant | 1.067 1.715 0.818 1.016  -3.563 | 0.477 0.474 0.519 0.482  0.530 | 4.997 13.088 2.482 4.448  45.278 | 1 1 1 1  1 | 0.025 <0.00 0.115 0.035  <0.001 | 2.906 5.555 2.266 2.761  0.028 | 1.141 2.194 0.819 1.074  --- | 7.405 14.064 6.268 7.096  --- |
| Step 6 | KSS_Expectation_ KOOS_Pain_ Age Constant | 1.065 1.907 1.198 -3.195 | 0.474 0.462 0.468 0.442 | 5.053 17.067 6.562 52.269 | 1 1 1 1 | 0.025 <0.001 0.010 <0.001 | 2.902 6.730 3.314 0.041 | 1.146 2.724 1.325 --- | 7.348 16.629 8.291 --- |
|  | **Test** |  |  | **χ^2^** | ***df*** | ***p-value*** |  |  |  |
|  | Overall model evaluation  Omnibus-test | | | 40.331 | 3 | <0.001 |  |  |  |
|  | Goodness-of-fit test  Hosmer-Lemeshow-test | | | 0.614 | 4 | 0.962 |  |  |  |

Full model Regression with “backward elimination”-method. Cox and Snell R² = 0.196; Nagelkerkes R² = 0.325.
BMI = Body mass index; CI = Confidence interval; df = Degrees of freedom; KOOS = Knee injury and Osteoarthritis Outcome Score; KSS = Knee Society Score; OR = Odds ratio, SE = Standard error; TKA = Total knee arthroplasty, VAS = Visual Analogue Scale.

**Supplementary Figure S1: Comparative analysis of receiver operating characteristic curves demonstrating the predictive value of predictor variables and** **risk score variations on patient satisfaction one year following TKA**

**
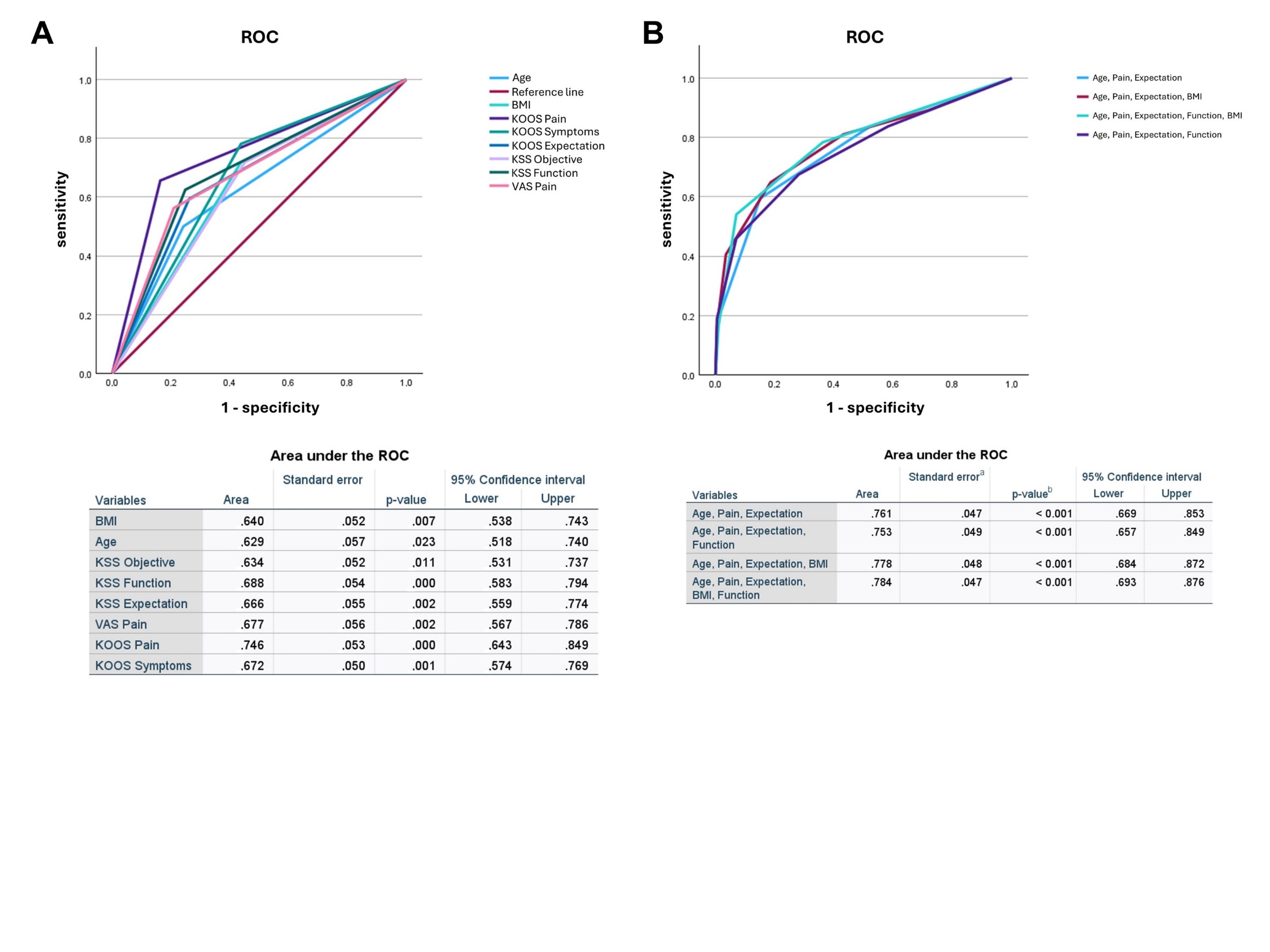
**

(A) Receiver Operating Characteristic (ROC) curves with Area Under the Curve (AUC) analyses of multiple single dichotomous predictors predicting dissatisfied patients; (B) ROC curves with corresponding AUC analyses of risk score variations. The risk score comprising Age, KOOS_Pain_, KSS_Expectation_, BMI, and KOOS_Function_ demonstrates the highest AUC, indicating superior predictive value for patients' dissatisfaction.
